# An Information Entropy-Based Approach for Computationally Identifying Histone Lysine Butyrylation

**DOI:** 10.3389/fgene.2019.01325

**Published:** 2020-02-14

**Authors:** Guohua Huang, Yang Zheng, Yao-Qun Wu, Guo-Sheng Han, Zu-Guo Yu

**Affiliations:** ^1^ Provincial Key Laboratory of Informational Service for Rural Area of Southwestern Hunan, Shaoyang University, Shaoyang, China; ^2^ Key Laboratory of Intelligent Computing and Information Processing of Ministry of Education and Hunan Key Laboratory for Computation and Simulation in Science and Engineering, Xiangtan University, Xiangtan, China; ^3^ School of Electrical Engineering and Computer Science, Queensland University of Technology, Brisbane, QLD, Australia

**Keywords:** butyrylation, random forest, histone, post-translational modification, information entropy

## Abstract

Butyrylation plays a crucial role in the cellular processes. Due to limit of techniques, it is a challenging task to identify histone butyrylation sites on a large scale. To fill the gap, we propose an approach based on information entropy and machine learning for computationally identifying histone butyrylation sites. The proposed method achieves 0.92 of area under the receiver operating characteristic (ROC) curve over the training set by 3-fold cross validation and 0.80 over the testing set by independent test. Feature analysis implies that amino acid residues in the down/upstream of butyrylation sites would exhibit specific sequence motif to a certain extent. Functional analysis suggests that histone butyrylation was most possibly associated with four pathways (systemic lupus erythematosus, alcoholism, viral carcinogenesis and transcriptional misregulation in cancer), was involved in binding with other molecules, processes of biosynthesis, assembly, arrangement or disassembly and was located in such complex as consists of DNA, RNA, protein, *etc*. The proposed method is useful to predict histone butyrylation sites. Analysis of feature and function improves understanding of histone butyrylation and increases knowledge of functions of butyrylated histones.

## Introduction

Butyrylation, a type of post-translation modification (PTM), refers to a biochemical interaction process where the butyryl functional group covalently modifies the lysine amino acid (Chen et al., 2007; Lu et al., 2018). Protein butyrylation is a newly discovered PTM (Chen et al., 2007). In the past 5 years, butyrylation's roles in the cellular process have been gradually uncovered. For example, Goudarzi et al. (2016) confirmed that histone butyrylation directly stimulates gene expression and inhibits Brdt Binding, Xu et al. (2018) found that butyrylation and acetylation are responsible for the phenotype and metabolic shifts of the endospore-forming *Clostridium acetobutylicum*, and Lu et al. (2018) revealed that butyrylation prefers poising gene activation by external stresses in the rise of submergence and starvation. Nevertheless, compared to such extensively-studied PTMs as acetylation (Kiemer et al., 2005; Basu et al., 2009; Gnad et al., 2010; Choudhary et al., 2014) and methylation (Chen et al., 2006; Shi et al., 2012b; Hamamoto et al., 2015; Shi et al., 2015; Wei et al., 2018), few functions of butyrylation are known. With in-depth exploration of butyrylation, more biological functions of butyrylation will undoubtedly be found.

Identifying butyrylation sites is an important foundation to further explore its functions. Biotechnologies whose representative is mass spectrometry provide a necessary approach to identify PTMs including butyrylation. Zhang et al. (2008) found four lysine butyrylation sites in histone yeast, Xu et al. (2014) 11 histone butyrylation sites in human cells, and Lu et al. (2018) identified four histone butyrylation sites in rice using mass spectrometry. Obviously, this strategy is not only labor-intensive and time-consuming, but also generally low-throughput. On the contrary, bioinformatics approaches provide an alternative to explore PTM sites, with characteristic being high-throughput. Since Hansen et al. (1995; 1998) proposed a method for computationally predicting mucin type O-glycosylation sites in the 1990s, dozens of computational approaches have been developed for identifying PTM sites (Blom et al., 2004; Xue et al., 2006; Zhou et al., 2006; Xu et al., 2008; Xu et al., 2010; Liu et al., 2011; Cai et al., 2012; Shi et al., 2012b; Zhang et al., 2012; Zhao et al., 2012; Xu et al., 2013; Zhang et al., 2013; Zhao et al., 2013; Huang et al., 2014; Shi et al., 2015; Xu et al., 2015a; Zhou et al., 2016). For instances, glycosylation identification includes the neural network-based method (Hansen et al., 1998), the support vector machine-based method (Li et al., 2006; Chen et al., 2008; Sasaki et al., 2009), the random forest-based method (Hamby and Hirst, 2008; Chuang et al., 2012), and ensemble learning algorithms (Caragea et al., 2007). Features used for predicting methylation sites are from protein sequences (Shao et al., 2009; Zhang et al., 2013; Qiu et al., 2014; Zhang et al., 2015; Wei et al., 2018), structure (Shien et al., 2009) or amino acid properties (Shi et al., 2012a). Xu et al. (2015b) proposed a pseudo amino acid composition-based method for predicting lysine succinylation. Zhou et al. (2004) proposed the GPS method for phosphorylation prediction, and Xu et al. (2008) proposed the method SUMOpre for sumoylation prediction. These computational methods are capable of screening potential modified sites on a large scale in a little time and help the former methods narrow the scope of verification of it. Here, we didn't plan to comprehensively review and discuss them, but propose a novel method based on information entropy and random forest for predicting histone butyryllysine. To the best of my knowledge, this is the first computational method for predicting butyrylation.

## Method and Materials

### Materials

One hundred butyrylated proteins were retrieved by searching both the Uniprot database (UniProt Consortium, 2018): https://www.uniprot.org/ and the Protein Lysine Modifications Database (PLMD): http://plmd.biocuckoo.org/ (Xu et al., 2017). The Uniprot database is a comprehensive repository of function annotation and sequences of proteins, which is updated every 2 months. The PLMD is dedicated to specifically collect lysine-modified proteins, and the current version 3.0 contains 284,780 modification events of 20 types of lysine-modified PTMs from 53501 proteins, including butyrylation, crotonylation and propionylation. Searching the Uniprot database with the keyword “butyryllysine”, we retrieved 91 butyrylated histones containing 317 butyrylation sites with the manual assertion. We downloaded the butyrylation data from the PLMD. Merging these two datasets and then removing abnormal proteins, we got 100 unique histones. To eliminate dependency of the computational method on homology, it is a general step to remove homology among protein sequences. The computational clustering tool (Huang et al., 2010) was used to cluster these 100 protein sequences with the sequence identity cut-off 0.7. Thirteen representative protein sequences were obtained among which sequence identity of any two is no more than 0.7. We selected six proteins from the Uniprot database as the training set which contained 17 butyrylation sites and the remaining seven from the PLMD as the testing set which contained nine butyrylation sites.

### Method

**Figure 1:**
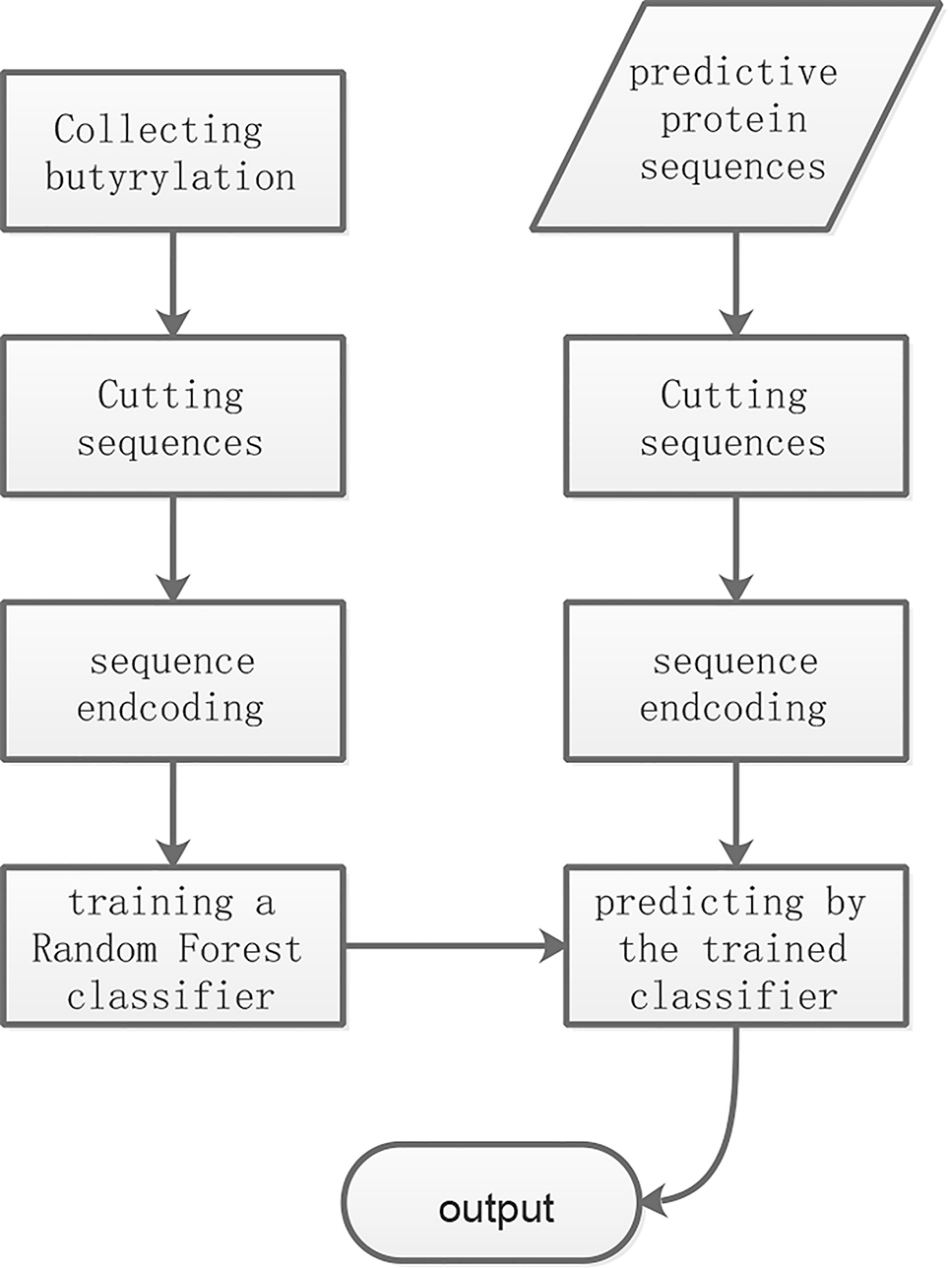
The flowchart of the proposed method.

As shown in **Figure 1**, the overall workflow of the proposed method consists mainly of four steps: cutting sequence, sequence encoding, training and predicting. The training and the predictive butyrylation histone sequences were cut into fragments which centered lysine with respectively N amino acid residues in the upstream and the downstream of it. That is, the window of (2N+1) residues centering lysine were separated out. For the windows containing lysine but less than 2N+1 residues, we prefixed or suffixed the character “X” to it for complement. The fragments undergoing butyrylation event were viewed as positive samples. We randomly selected 18 non-butyrylation fragments from the training set as training negative samples, and 18 non-butyrylation ones from the testing set as the testing negative samples. The **Supplementary Table 1** listed all the training and the testing butyrylation as well as the non-butyrylation sites. For each fragment with (2N+1) resides, the information entropy-based encoding (IEE) and the composition of k-space amino acid pair (CKSAAP) transformed it into numerical feature. After the random forest algorithm trained a classifier using the training set with the numerical features, the unknown protein sequences were input into the trained classifier for final prediction.

#### IEE

Histone butyrylation is assumed as a stochastic system described as P*^i^*(α) which stands for probability of the amino acid α occurring at the i-th position. Obviously, P*^i^*(α) is an *m*-by-*n* matrix where *m* is the number of characters of amino acid (here *m* is 21) and *n* the length of the sequence (here *n*=2*N+1). This stochastic system is measured by the information entropy of amino acid (PIEA) and the information entropy of position (PIEP), which are denoted respectively by

(1) $$\text{PIEA}\left(\alpha \right)=\sum_{i=1}^{2N+1}-P^{i}\left(\alpha \right)\log P^{i}\left(\alpha \right)$$

and

(2) $$\text{PIEP}\left(i\right)=\sum_{\alpha \in \Phi }-P^{i}\left(\alpha \right)\log P^{i}\left(\alpha \right)$$

where Φ represents the set of characters of amino acid. P*^i^*(α) can be estimated by calculating frequencies of amino acid over all the positive samples in the training set, respectively. The PIEA and the PIEP represent uncertainty of the butyrylation system. The more the PIEA and the PIEP are, the more uncertainty the system is. After a new sample *s* was added to the system, its information entropies of amino acid and position are denoted by PIEPs and PIEAs. The variation of information entropies after addition of the new sample to the system is defined by

(3) PVIEA=PIEA(α)−PIEAs(α)

and

(4) PVIEP=PIEP(i)−PIEPs(i).

Similarly, the non-butyrylation system is also assumed as a distinct stochastic system N*^i^*(α) which is estimated by calculating frequencies of amino acid over all the negative samples in the training set, respectively. The information entropies of amino acid (NIEA) and the information entropies of position (NIEP) for the non-butyrylation system are defined by

(5) $$\text{NIEA}\left(\alpha \right)=\sum_{i=1}^{2N+1}-N^{i}\left(\alpha \right)\log N^{i}\left(\alpha \right)$$

and

(6) $$\text{NIEP}\left(i\right)=\sum_{\alpha \in \Phi }-{\text{N}}^{i}\left(\alpha \right)\log N^{i}\left(\alpha \right)$$

The variation of information entropies after addition of the new sample *s* to the non-butyrylation system is defined by

(7) NVIEA=NIEA(α)−NIEAs(α)

and

(8) NVIEP=NIEP(i)−NIEPs(i),

where NIEAs and NIEPs denote respectively information entropies of amino acid and position after addition of the new sample to the non-butyrylation system. The new sample is encoded by PVIEA-NVIEA and PVIEP-NVIEP. Therefore, for each sample, we obtain (21 + 2N+1) feature to represent it.

#### CKSAAP

The CKSAAP is occurrence frequency of *k*-spaced amino acid pair which is spaced by up to *k* residues. *k* is equal to or more than 0. For example, AA, AC, ..., YX and XX belong to 0-spaced amino acid pair, while AA, AC, ...., XX, ABA, ABC, ..., and XBX to 1-spaced amino acid pair. Generally, there are (K+1)*21*21 features for k-spaced amino acid pair. The CKSAAP were widely applied to prediction of phosphorylation, methylation, palmitoylation, pupylation, ubiquitination and O-glycosylation (Chen et al., 2008; Wang et al., 2009; Chen et al., 2011; Zhao et al., 2012; Tung, 2013; Zhang et al., 2013).

#### Feature Normalization

All the features are normalized by the following formula

(9) $${\text{X}}_{k}^{n}=\frac{x_{k}^{n}-\underset{m}{\min}\left\{x_{k}^{m}\right\}}{\underset{m}{\max}\left\{x_{k}^{m}\right\}-\underset{m}{\min}\left\{x_{k}^{m}\right\}},$$

where *x_k_^n^* denotes the k-th non-normalized feature of the sample *n*. The normalized feature lies between 0 and 1.

#### Random Forest

Random forest by Breiman (2001) is an ensemble learning algorithm which combines decision trees for vote. The random forest is composed mainly of constructing of decision trees and voting over all the decision trees for the given sample. Each decision tree grow out of the new training set drawn with replacement from the training set and with *m* << *M* randomly selected features (*M* is the total number of sample features). The majority of vote for a sample is the output class for classification. The advantage of Random forests is that it overcome overfitting which occurred in decision trees, and meanwhile produce a limiting value of the generalization error. For more details of random forest, readers can refer to relevant references. Here, we use Weka software package (Hall et al., 2009) which realized a wide range of machine learning algorithms using the Java programming language.

## Cross Validation And Metrics

We used 3-fold cross validation to examine performance of the proposed method. For 3-fold cross validation, *n* training samples are divided into three parts in approximate or equal size. Each part is in turn used as the testing set which is predicted by the trained classifier over the other two parts. Independent test was used to examine generalization ability of the proposed method.

The receiver operating characteristic (ROC) curve was used to assess the predictive performances, which is plotting true positive rate against false positive rate under various threshold. Area under the ROC curve (AUC) was used to compare it, ranging from 0 to 1. The AUC was 1, meaning the perfect prediction, while the AUC was 0.5, indicating the uninformative classifier.

## Results and Discussion

**Figure 2:**
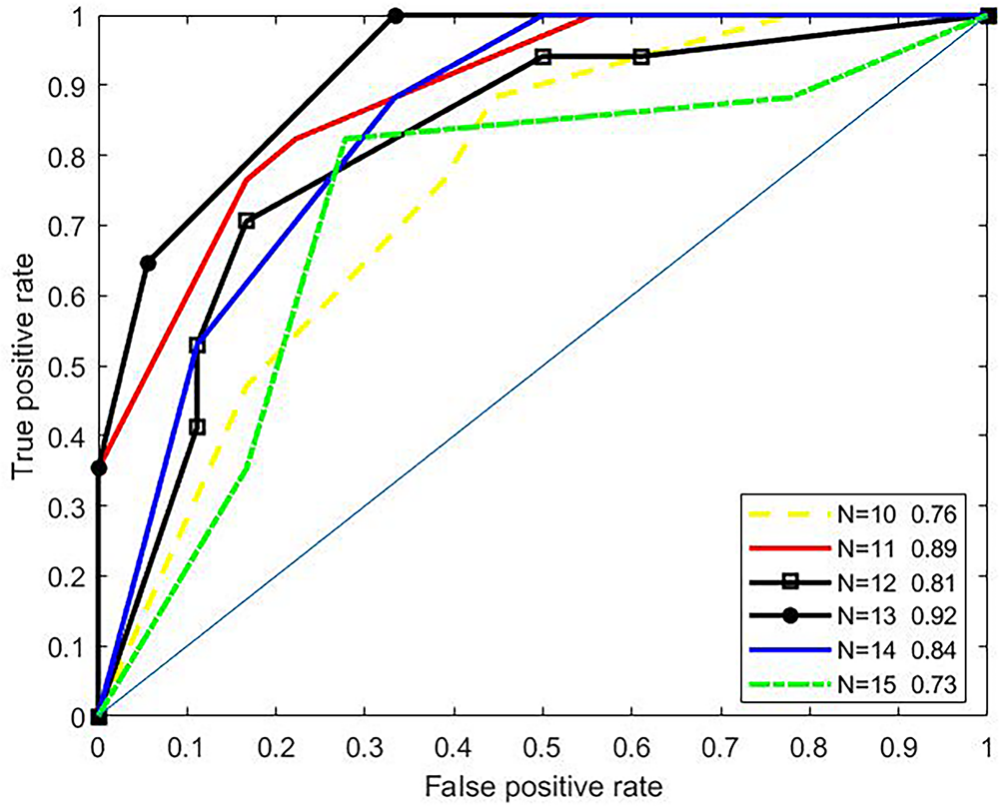
ROC curves under various parameter *N*.

To investigate effects of the parameter *N* (length of amino acid residues in the upstream or the downstream of the butyrylation sites) on the predictive performances, we conducted 3-fold cross validation over the training set. Most approaches for predicting PTM sites generally set *N* to the interval of 10 to 15 (Hou et al., 2014; Huang et al., 2014; Xu et al., 2015a; Hasan et al., 2016; Jia et al., 2016a; Jia et al., 2016b; Xu et al., 2016; Wang et al., 2017). For example, the iSulf-Cys for predicting s-sulfenylation sites (Xu et al., 2016) adopted a window of 21 residues (i.e., *N*=10), while the iSuc-PseOpt (Jia et al., 2016a), a tool for predicting lysine succinylation sites, used *N*=15 amino acid residues of the upstream/downstream of the modified site. Therefore, we tested *N* only between 10 to 15. As shown in **Figure 2**, the ROC curves of 3-fold cross validation under various *N* were plotted. The best AUC (N=13) is 0.92, while the worst (*N*=15) is 0.73. Therefore, we set N to 13.

**Figure 3:**
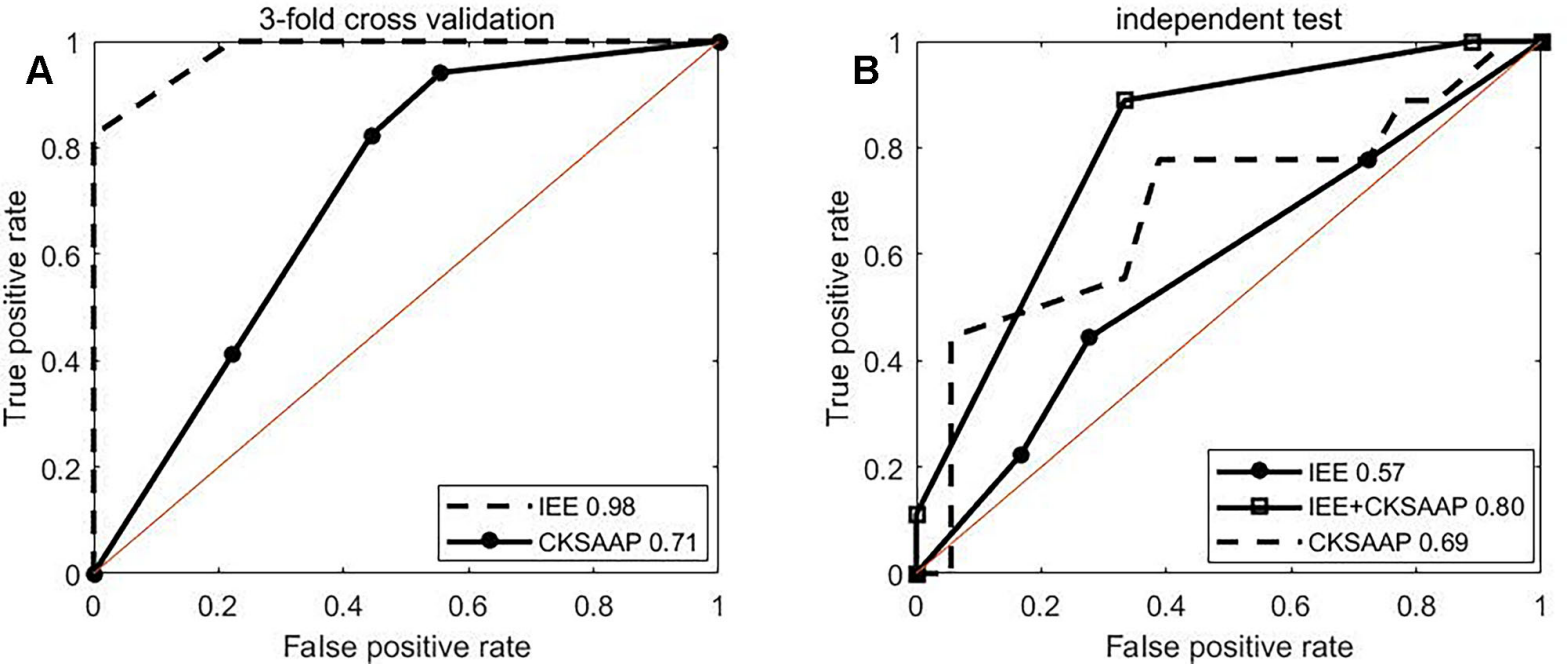
ROC curves. **(A**, **B)** depict ROC curves of 3-fold cross validation and independent test, respectively.

ROC curves of 3-fold cross validation over the training set for single type of IEE and for single type of CKSAAP features were shown in **Figure 3A**. The IEE outperformed the CKSAAP and the combination of two. ROC curves of independent test were plotted in **Figure 3B**. Obviously, the combination performs best, followed by the CKSAAP and then by the IEE feature. The single performance of the IEE feature is best over the training set, but worst over the testing set. The single performance of the CKSAAP is worst over the training set. The combination of IEE and CKSAAP features performs most stable, with 0.92 of AUC over 3-fold cross validation and 0.80 of AUC over independent test respectively.

### Analysis Of Sequence Pattern

**Figure 4:**
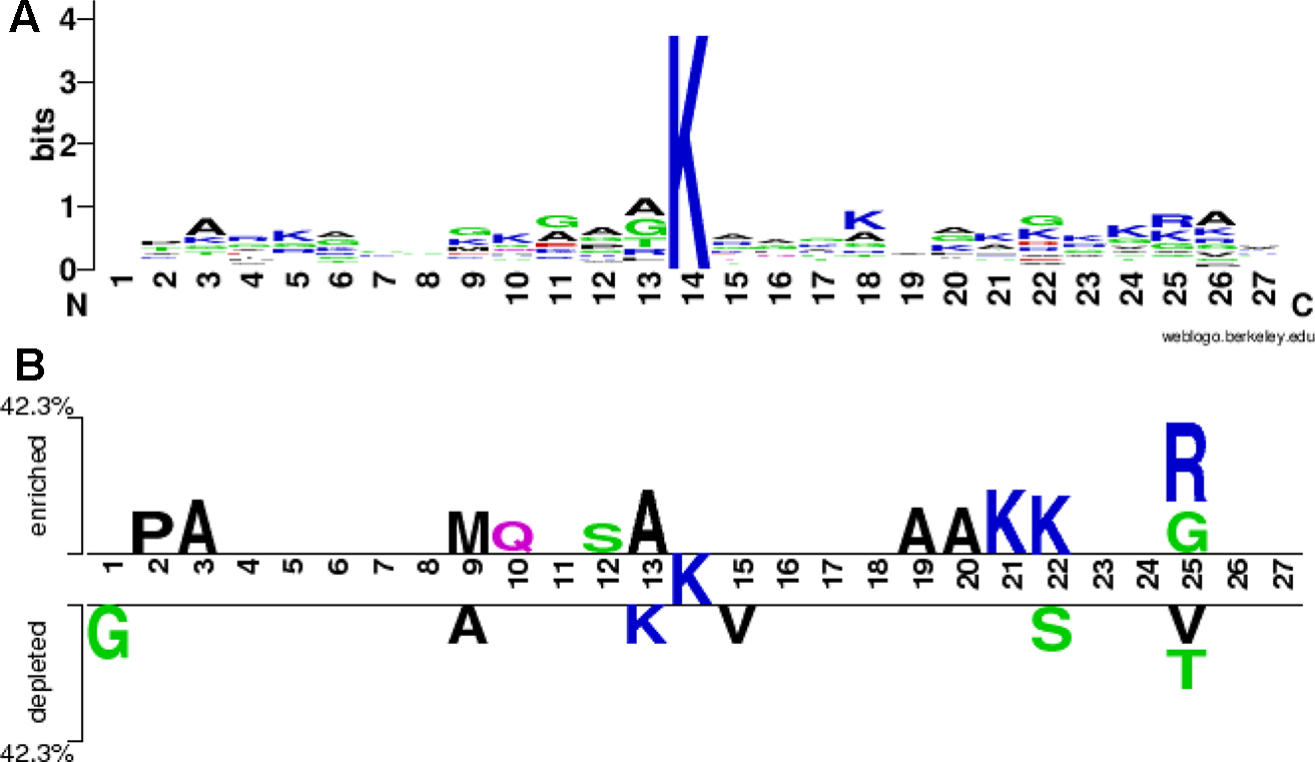
Sequence logo. **(A)** is sequence logo of all the positive samples and **(B)** is sequence logo of all the positive and the negative samples.

We used the WebLogo program (Crooks et al., 2004) to draw a sequence logo of all the 26 positive samples both from the training and the testing sets, as shown in **Figure 4A**. The stacks at the positions 13, 25 and 26 is higher, followed by the positions 22, 18 and 11, indicating that these positions would be more evolutionarily conservative. On the contrary, the stacks at the positions 1, 7, 8 and 19 is lower, implying these positions would be less conservative. The symbols A (alanine) at the positions 3, 6, 12, 13, and 26, K (lysine) at the positions 5, 10, 18, 21 and 24, G (glycine) at the positions 9, 11 and 22, and R (arginine) at the position 25 are higher at respective stack, indicating that these amino acids alanine, lysine, glycine and arginine would appear more frequently at these corresponding positions. The two-sample sequence logo was plotted using a web-based software (Vacic et al., 2006) http://www.twosamplelogo.org/index.html. The positive samples were 26 non-redundant fragments containing butyrylation sites, while the negative ones were 36 fragments, 62 in total. In comparison to previous single-sample sequence logo, the two-sample logo more intuitively exhibited statistically significant differential residues between two classes. As shown in **Figure 4B**, the symbols K at these positions 21 and 22, A at these positions 3, 13,19 and 20, P (proline) at the position 2, M (methionine) at the position 9, Q (glutamine) at the position 10, S (serine) at the position 12, G and R at the position 25, were enriched in the butyrylation fragments, while G at the position 1, A at the position 9, K at the position 13, S at the position 22, V (valine) at the positions 15 and 25, and T (threonine) at the position 25 were depleted. Combining the information from **Figures 4A, B**, we speculated that alanine at the position 3 and 13, lysine at the position 21 and arginine at the position 25 would be associated with histone butyrylation.

### Analysis of Information Entropy Feature

**Figure 5:**
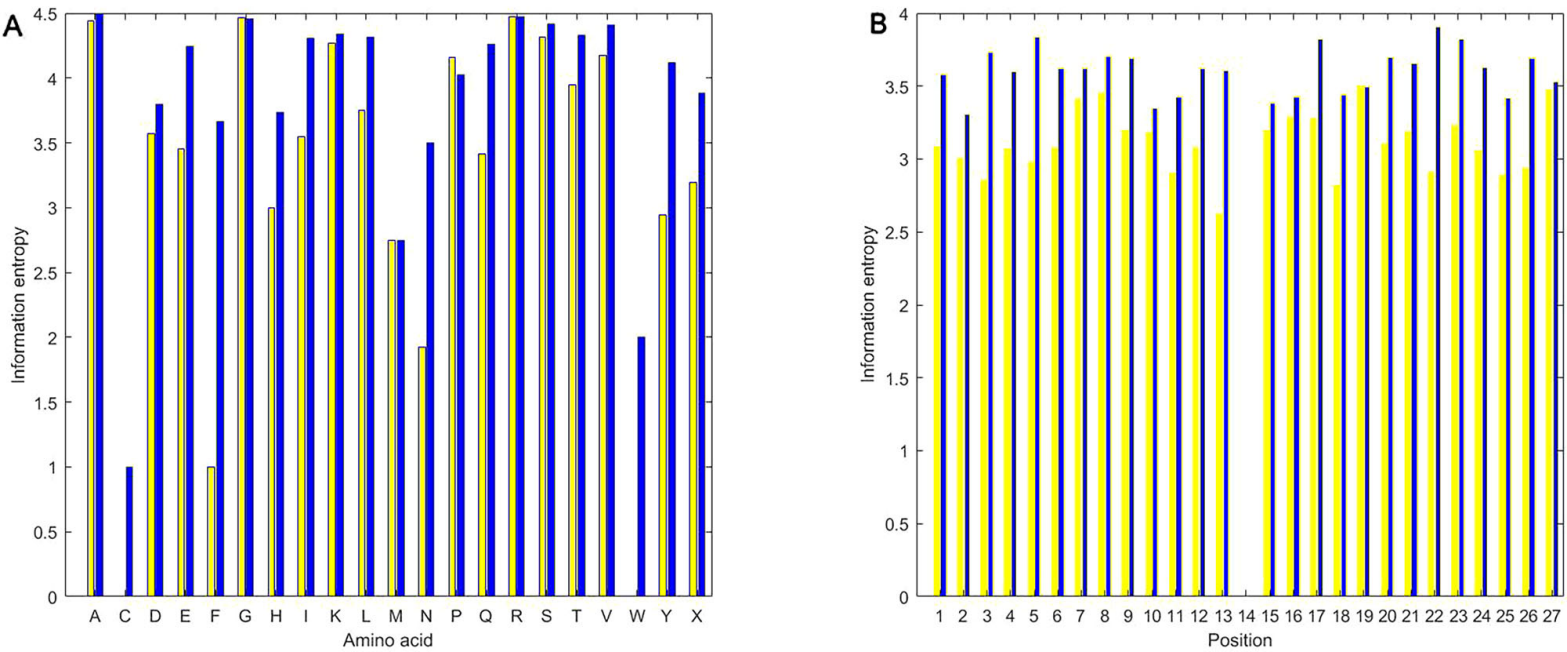
Information entropies. **(A)** represents information entropies of PIEA and NIEA. **(B)** represents information entropies of PIEP and NIEP.

As shown in **Figure 5**, we calculated information entropies of all the used positive and the negative samples in the experiment using the equations (1) and (2). Regardless of amino acid or position, information entropies of butyrylation wholly are less than those of non-butyrylation, indicating that the distribution of amino acid followed more a rule in the butyrylation than at random. The information entropies of C (cysteine) and W (tryptophan) are near or equal to zero (**Figure 5A**), implying that two types of amino acid would occur in a fixed way not at random. The information entropies of F (phenylalanine) and N (asparagine) are much less in the butyrylation than in the non-butyrylation, indicating that phenylalanine and asparagine would play a role in the butyrylation. Information entropies of G, P, M and R in the positive sample is approximately equal or more than those in the negative samples, respectively. This indicated non-difference of these amino acids between butyrylation and non-butyrylation. The information entropies of position in the butyrylation is less than those in the non-butyrylation exception the position 14 (**Figure 5B**), indicating that amino acid distribution in the butyrylation would follow more rules than at random.

### Analysis of CKSAAP Feature

**Figure 6:**
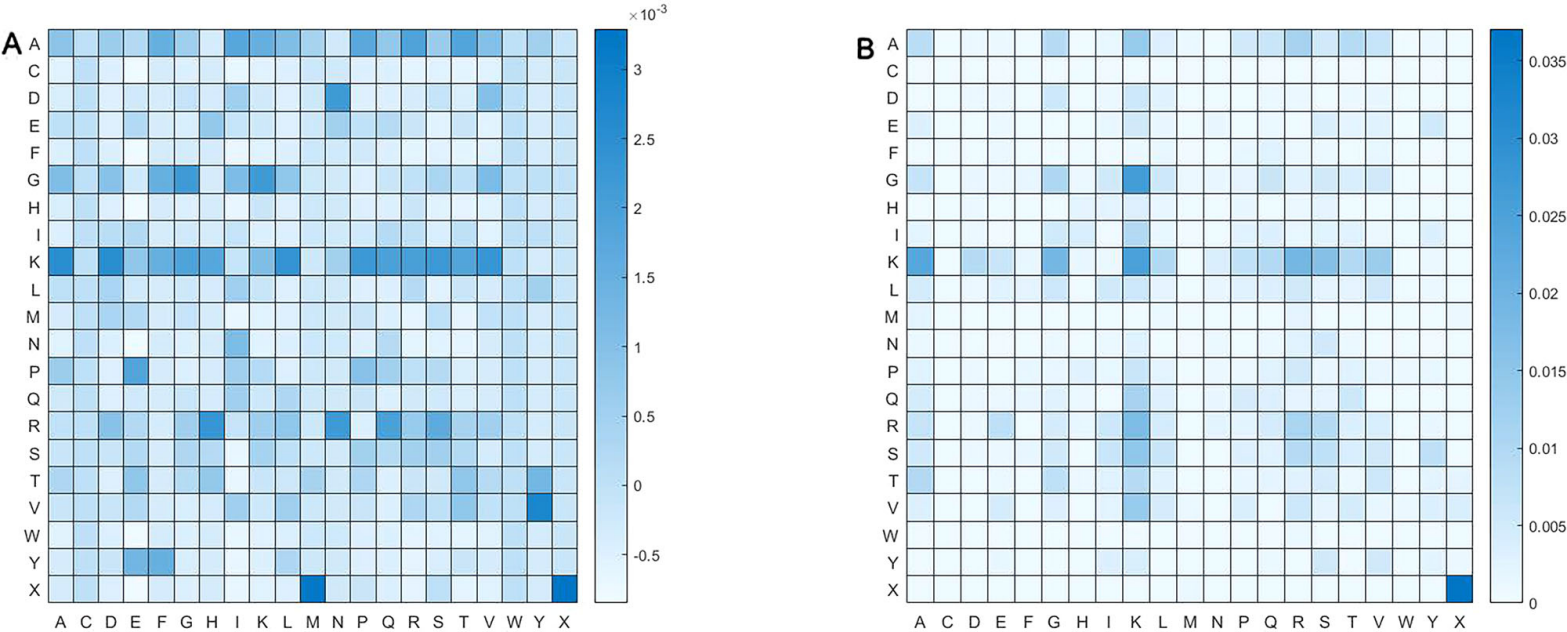
Heatmap of amino acid pair. **(A)** represents heatmap of all the positive samples and **(B)** heatmap of all the negative samples.

We calculated pairs of amino acid separated by up to one residue. Namely, amino acid pair might be of such form as αβ and αΔβ, where Δ represent an amino acid. **Figure 6** shows frequency of pair of amino acid. Obviously, distribution of amino acid pairs in the butyrylation differs largely from that in the non-butyrylation. The butyrylation focuses mainly on these amino acid pairs of DN, GG, GK, KA, KD, KL, KP, KS, KV, PE, RH, RN, VY, XM and XX, while the non-butyrylation on GK, KA, KK and XX.

### Analysis of Function for Histone Butyrylation

We used the PANTHER classification system (Mi et al., 2013) (http://www.pantherdb.org/) for functional analysis of histone butyrylation. Both statistical over-representation tests of *Homo sapiens* butyrylation histones against the whole *H. sapiens* genes and of *Mus musculus* butyrylation histones against the whole *M. musculus* genes were performed. The significantly over-represented GO terms (*P *< 0.05) for biological process, molecular function and cellular composition are listed in **Supplementary Table 2–7**. It is obviously observed that all GO terms of *M. musculus* butyrylated histones appeared in the *H. sapiens* histones, except cytosol (GO:0005829) which is defined as the part of the cytoplasm which does not contain organelles but contain such particulate matter as protein complexes. However, some GO terms of *H. sapiens* butyrylated histones fail to fall into the set of GO terms of *M. musculus* histones. For example, in terms of molecular function, STAT family protein binding (GO:0097677), RNA polymerase II core promoter sequence-specific DNA binding (GO:0000979), core promoter sequence-specific DNA binding (GO:0001046), core promoter binding (GO:0001047), chromatin binding (GO:0003682), protein-containing complex binding (GO:0044877), protein binding (GO:0005515) and binding (GO:0005488) are significant over-represented GO terms in *H. sapiens* butyrylation histone, not in *M. musculus* histones. The difference of the first four molecular functions between two species would be caused by the small-sample question. The number of studied *M. musculus* butyrylated histones is 17, less than the number of *H. sapiens* histones. The term GO:0097677 appeared two times, and these three terms GO:0000979, GO:0001046 and GO:0001047 appeared three times in these 30 butyrylated *H. sapiens* histones, while they would likely appear less than two times in these 17 butyrylated *M. musculus* histones. Only functions appearing two times or more would be statistically analyzed. Therefore, these four molecular functions could not separate *H. sapiens* from *M. musculus* histones. GO:0044877 appeared 10 times, GO:0003682 11 times, GO:0005515 29 times, while GO:0005488 appeared 30 times in the *H. sapiens* histone. It is rational to infer occurring more than two times in 17 *M. musculus* butyrylation histones, but they were not significant over-represented GO terms. This indicated that these later four molecular functions were enriched only in the *H. sapiens*, not in all the species.

**Table 1** listed the most significant five GO terms of molecular function, biological process and cellular component in the butyrylated *H. sapiens* histone which all belonged to the set of the over-represent GO terms in the *M. musculus* histones respectively. These three terms GO:0003677 (DNA binding), GO:0003676 (nucleic acid binding) and GO:0031492 (nucleosomal DNA binding) are defined as interacting selectively and non-covalently with DNA, with any nucleic acid and with the DNA portion of a nucleosome, respectively. GO:0046982 (protein heterodimerization activity) is defined as interacting selectively and non-covalently with a non-identical protein to form a heterodimer, whose relationship with GO:0046983 (protein dimerization activity) is “is a”. All the five terms belongs to the ancestor GO:0005488 (binding) via the “is a” relationship, implying that butyrylation histones could bind other molecules such as DNA, nucleic acid or protein. GO:0006334 (nucleosome assembly) is defined as the aggregation, arrangement and bonding together of a nucleosome, the beadlike structural units of eukaryotic chromatin composed of histones and DNA, which is of “is a” relationship with GO:0034728 (nucleosome organization) and of “part of ” relationship with GO:0031497 (chromatin assembly). The term GO:0031497 is of “part of” relationship with GO:0006323 (DNA packaging) and of “is a” relationship with GO:0006333 (chromatin assembly or disassembly). These five terms finally are traced up to two terms: GO:0016043 (cellular component organization) and GO:0044085 (cellular component biogenesis), indicating that butyrylation histones might be associated with these processes of biosynthesis, assembly, arrangement or disassembly. The term GO:0000786 (nucleosome) refers to a complex consisting of DNA wound around a multi-subunit core and associated proteins, which forms the primary packing unit of DNA into higher order structures. The term GO:0000786 is of “is a” relationships both with the term GO:0044815 (DNA packaging complex) and with GO:0032993 (protein-DNA complex) and is of “part of” relationship with the term GO:0000785 (chromatin) which is of part of relationship with the term GO:0005694 (chromosome). These results indicate that butyrylation histone might be located in a complex composed of DNA, proteins, *etc*.

**Table 1:** Most significant five GO terms of molecular function, biological process and cellular component for *Homo sapiens*.

Molecular function	Biological process	Cellular component
Protein heterodimerization activity (GO:0046982)	Nucleosome assembly (GO:0006334)	Nucleosome (GO:0000786)
DNA binding (GO:0003677)	Chromatin assembly (GO:0031497)	DNA packaging complex (GO:0044815)
Protein dimerization activity (GO:0046983)	Chromatin assembly or disassembly (GO:0006333)	Protein-DNA complex (GO:0032993)
Nucleic acid binding (GO:0003676)	Nucleosome organization (GO:0034728)	Chromatin (GO:0000785)
Nucleosomal DNA binding (GO:0031492)	DNA packaging (GO:0006323)	Chromosome (GO:0005694)

We used the *David* (Database for Annotation, Visualization and Integrated Discovery) (Huang da et al., 2009a; Huang da et al., 2009b) to explore biological pathways in which the butyrylated histones are potential to be involved. The *David* is one of most popular tool for enrichment analysis of gene function, currently including over 40 annotation categories, such as ordinary GO terms, protein functional domains, bio-pathways, *etc*. The backgrounds for *H. sapiens* and *M. musculus* butyrylation histones were respectively the whole *H. sapiens* and the whole *M. musculus* genes. The statistically significant Kyoto Encyclopedia of Genes and Genomes (KEGG) pathways (*P*-value < 0.01) are systemic lupus erythematosus, alcoholism, viral carcinogenesis and transcriptional misregulation in cancer, whether for *H. sapiens* or for *M. musculus* genes, indicating that histone butyrylation is involved in similar bio-pathway.

## Conclusion

Histone butyrylation is a newly discovered PTM, whose mechanism remains unknown. In this paper, we presented an approach based on information entropy and machine learning for identifying histone butyrylation sites. To the best of our knowledge, this is the first computational method for identifying histone butyrylation sites. By comparing sequences, IEE and CKSAAP between butyrylation and non-butyrylation, we found some specific characteristics implying potential and hidden pattern of histone butyrylation. The statistical test suggests that the butyrylation histone might be of binding with other molecules, be associated with the processes of biosynthesis, assembly, arrangement or disassembly, be located in the complex of DNA, protein, *etc*, and be involved in the such pathway as systemic lupus erythematosus, alcoholism, viral carcinogenesis and transcriptional misregulation in cancer.

## Data Availability Statement

Butyrylated proteins were retrieved by searching both the Uniprot database(UniProt Consortium, 2018): https://www.uniprot.org/ and the Protein Lysine Modifications Database: http://plmd.biocuckoo.org/_ (P68432; P62804; P62807;P02294; Q6DN03; Q3SZB8; O75367; P0C0S5; P16104; Q02539; Q75WM6; Q8IZA3; Q92522).

## Author Contributions

GH, YZ and Z-GY conceived the method. GH and YZ collected data. GH and YZ performed the experiment. GH, YZ, Y-QW, G-SH and Z-GY analyzed the results and wrote the manuscript. All the authors read the manuscript and approved the final manuscript.

## Funding

This work is supported by National Natural Science Foundation of China (61672356, 11871061), by Scientific Research Fund of Hunan Provincial Education Department (18A394), and by the open project of Hunan Key Laboratory for Computation and Simulation in Science and Engineering (2019LCESE03).

## Conflict of Interest

The authors declare that the research was conducted in the absence of any commercial or financial relationships that could be construed as a potential conflict of interest.

## Supplementary Material

The Supplementary Material for this article can be found online at: https://www.frontiersin.org/articles/10.3389/fgene.2019.01325/full#supplementary-material

## References

[B1] Basu A. Rose K. L. Zhang J. Beavis R. C. Ueberheide B. Garcia B. A. (2009). Proteome-wide prediction of acetylation substrates. Proc. Natl. Acad. Sci. U. S. A. 106 (33), 13785–13790. 10.1073/pnas.0906801106 19666589 PMC2728972

[B2] Blom N. Sicheritz-Ponten T. Gupta R. Gammeltoft S. Brunak S. (2004). Prediction of post-translational glycosylation and phosphorylation of proteins from the amino acid sequence. Proteomics 4 (6), 1633–1649. 10.1002/pmic.200300771 15174133

[B3] Breiman L. (2001). Random forests. Mach. Learn. 45 (1), 5–32. 10.1023/A:1010933404324

[B4] Cai Y. Huang T. Hu L. Shi X. Xie L. Li Y. (2012). Prediction of lysine ubiquitination with mRMR feature selection and analysis. Amino Acids 42 (4), 1387–1395. 10.1007/s00726-011-0835-0 21267749

[B5] Caragea C. Sinapov J. Silvescu A. Dobbs D. Honavar V. (2007). Glycosylation site prediction using ensembles of support vector machine classifiers. BMC Bioinf. 8, 438. 10.1186/1471-2105-8-438 PMC2220009 17996106

[B6] Chen H. Xue Y. Huang N. Yao X. Sun Z. (2006). MeMo: a web tool for prediction of protein methylation modifications. Nucleic Acids Res. 34 (Web Server issue), W249–W253. 10.1093/nar/gkl233 16845004 PMC1538891

[B7] Chen Y. Sprung R. Tang Y. Ball H. Sangras B. Kim S. C. (2007). Lysine propionylation and butyrylation are novel post-translational modifications in histones. Mol. Cell Proteomics 6 (5), 812–819. 10.1074/mcp.M700021-MCP200 17267393 PMC2911958

[B8] Chen Y. Z. Tang Y. R. Sheng Z. Y. Zhang Z. (2008). Prediction of mucin-type O-glycosylation sites in mammalian proteins using the composition of k-spaced amino acid pairs. BMC Bioinf. 9, 101. 10.1186/1471-2105-9-101 PMC2335299 18282281

[B9] Chen Z. Chen Y. Z. Wang X. F. Wang C. Yan R. X. Zhang Z. (2011). Prediction of ubiquitination sites by using the composition of k-spaced amino acid pairs. PloS One 6 (7), e22930. 10.1371/journal.pone.0022930 21829559 PMC3146527

[B10] Choudhary C. Weinert B. T. Nishida Y. Verdin E. Mann M. (2014). The growing landscape of lysine acetylation links metabolism and cell signalling. Nat. Rev. Mol. Cell Biol. 15 (8), 536–550. 10.1038/nrm3841 25053359

[B11] Chuang G.-Y. Boyington J. C. Joyce M. G. Zhu J. Nabel G. J. Kwong P. D. (2012). Computational prediction of N-linked glycosylation incorporating structural properties and patterns. Bioinformatics 28 (17), 2249–2255. 10.1093/bioinformatics/bts426 22782545 PMC3426846

[B12] Crooks G. E. Hon G. Chandonia J.-M. Brenner S. E. (2004). WebLogo: a sequence logo generator. Genome Res. 14 (6), 1188–1190. 10.1101/gr.849004 15173120 PMC419797

[B13] Gnad F. Ren S. Choudhary C. Cox J. Mann M. (2010). Predicting post-translational lysine acetylation using support vector machines. Bioinformatics 26 (13), 1666–1668. 10.1093/bioinformatics/btq260 20505001 PMC2887055

[B14] Goudarzi A. Zhang D. Huang H. Barral S. Kwon O. K. Qi S. (2016). Dynamic competing histone H4 K5K8 acetylation and butyrylation are hallmarks of highly active gene promoters. Mol. Cell 62 (2), 169–180. 10.1016/j.molcel.2016.03.014 27105113 PMC4850424

[B15] Hall M. Frank E. Holmes G. Pfahringer B. Reutemann P. Witten I. H. (2009). The WEKA data mining software: an update. ACM SIGKDD Explor. Newsl. 11 (1), 10–18. 10.1145/1656274.1656278

[B16] Hamamoto R. Saloura V. Nakamura Y. (2015). Critical roles of non-histone protein lysine methylation in human tumorigenesis. Nat. Rev. Cancer 15 (2), 110–124. 10.1038/nrc3884 25614009

[B17] Hamby S. E. Hirst J. D. (2008). Prediction of glycosylation sites using random forests. BMC Bioinf. 9, 500. 10.1186/1471-2105-9-500 PMC2651179 19038042

[B18] Hansen J. E. Lund O. Engelbrecht J. Bohr H. Nielsen J. O. (1995). Prediction of O-glycosylation of mammalian proteins: specificity patterns of UDP-GalNAc: polypeptide N-acetylgalactosaminyltransferase. Biochem. J. 308, 801–813. 10.1042/bj3080801 8948436 PMC1136796

[B19] Hansen J. E. Lund O. Tolstrup N. Gooley A. A. Williams K. L. Brunak S. (1998). NetOglyc: prediction of mucin type O-glycosylation sites based on sequence context and surface accessibility. Glycoconj. J. 15 (2), 115–130. 10.1023/a:1006960004440 9557871

[B20] Hasan M. M. Yang S. Zhou Y. Mollah M. N. (2016). SuccinSite: a computational tool for the prediction of protein succinylation sites by exploiting the amino acid patterns and properties. Mol. Biosyst. 12 (3), 786–795. 10.1039/c5mb00853k 26739209

[B21] Hou T. Zheng G. Zhang P. Jia J. Li J. Xie L. (2014). LAceP: lysine acetylation site prediction using logistic regression classifiers. PloS One 9 (2), e89575. 10.1371/journal.pone.0089575 24586884 PMC3930742

[B22] Huang da W. Sherman B. T. Lempicki R. A. (2009a). Bioinformatics enrichment tools: paths toward the comprehensive functional analysis of large gene lists. Nucleic Acids Res. 37 (1), 1–13. 10.1093/nar/gkn923 19033363 PMC2615629

[B23] Huang da W. Sherman B. T. Lempicki R. A. (2009b). Systematic and integrative analysis of large gene lists using DAVID bioinformatics resources. Nat. Protoc. 4 (1), 44–57. 10.1038/nprot.2008.211 19131956

[B24] Huang Y. Niu B. Gao Y. Fu L. Li W. (2010). CD-HIT Suite: a web server for clustering and comparing biological sequences. Bioinformatics 26 (5), 680–682. 10.1093/bioinformatics/btq003 20053844 PMC2828112

[B25] Huang G. Lu L. Feng K. Zhao J. Zhang Y. Xu Y. (2014). Prediction of S-nitrosylation modification sites based on kernel sparse representation classification and mRMR algorithm. BioMed. Res. Int. 2014, 438341. 10.1155/2014/438341 25184139 PMC4145740

[B26] Jia J. Liu Z. Xiao X. Liu B. Chou K. C. (2016a). iSuc-PseOpt: identifying lysine succinylation sites in proteins by incorporating sequence-coupling effects into pseudo components and optimizing imbalanced training dataset. Anal. Biochem. 497, 48–56. 10.1016/j.ab.2015.12.009 26723495

[B27] Jia J. Liu Z. Xiao X. Liu B. Chou K. C. (2016b). pSuc-Lys: predict lysine succinylation sites in proteins with PseAAC and ensemble random forest approach. J. Theor. Biol. 394, 223–230. 10.1016/j.jtbi.2016.01.020 26807806

[B28] Kiemer L. Bendtsen J. D. Blom N. (2005). NetAcet: prediction of N-terminal acetylation sites. Bioinformatics 21 (7), 1269–1270. 10.1093/bioinformatics/bti130 15539450

[B29] Li S. Liu B. Zeng R. Cai Y. Li Y. (2006). Predicting O-glycosylation sites in mammalian proteins by using SVMs. Comput. Biol. Chem. 30 (3), 203–208. 10.1016/j.compbiolchem.2006.02.002 16731044

[B30] Liu Z. Cao J. Ma Q. Gao X. Ren J. Xue Y. (2011). GPS-YNO2: computational prediction of tyrosine nitration sites in proteins. Mol. Biosyst. 7 (4), 1197–1204. 10.1039/c0mb00279h 21258675

[B31] Lu Y. Xu Q. Liu Y. Yu Y. Cheng Z. Y. Zhao Y. (2018). Dynamics and functional interplay of histone lysine butyrylation, crotonylation, and acetylation in rice under starvation and submergence. Genome Biol. 19 (1), 144. 10.1186/s13059-018-1533-y 30253806 PMC6154804

[B32] Mi H. Muruganujan A. Casagrande J. T. Thomas P. D. (2013). Large-scale gene function analysis with the PANTHER classification system. Nat. Protoc. 8 (8), 1551–1566. 10.1038/nprot.2013.092 23868073 PMC6519453

[B33] Qiu W. R. Xiao X. Lin W. Z. Chou K. C. (2014). iMethyl-PseAAC: identification of protein methylation sites via a pseudo amino acid composition approach. BioMed. Res. Int. 2014, 947416. 10.1155/2014/947416 24977164 PMC4054830

[B34] Sasaki K. Nagamine N. Sakakibara Y. (2009). Support vector machine prediction of N-and O-glycosylation sites using whole sequence information and subcellular localization. IPSJ Trans. Bioinf. 2, 25–35. 10.2197/ipsjtbio.2.25

[B35] Shao J. Xu D. Tsai S. N. Wang Y. Ngai S. M. (2009). Computational identification of protein methylation sites through bi-profile Bayes feature extraction. PloS One 4 (3), e4920. 10.1371/journal.pone.0004920 19290060 PMC2654709

[B36] Shi S. P. Qiu J. D. Sun X. Y. Suo S. B. Huang S. Y. Liang R. P. (2012a). PLMLA: prediction of lysine methylation and lysine acetylation by combining multiple features. Mol. Biosyst. 8 (5), 1520–1527. 10.1039/c2mb05502c 22402705

[B37] Shi S. P. Qiu J. D. Sun X. Y. Suo S. B. Huang S. Y. Liang R. P. (2012b). PMeS: prediction of methylation sites based on enhanced feature encoding scheme. PloS One 7 (6), e38772. 10.1371/journal.pone.0038772 22719939 PMC3376144

[B38] Shi Y. Guo Y. Hu Y. Li M. (2015). Position-specific prediction of methylation sites from sequence conservation based on information theory. Sci. Rep. 5, 12403. 10.1038/srep12403 26202727 PMC5378888

[B39] Shien D. M. Lee T. Y. Chang W. C. Hsu J. B. Horng J. T. Hsu P. C. (2009). Incorporating structural characteristics for identification of protein methylation sites. J. Comput. Chem. 30 (9), 1532–1543. 10.1002/jcc.21232 19263424

[B40] Tung C. W. (2013). Prediction of pupylation sites using the composition of k-spaced amino acid pairs. J. Theor. Biol. 336, 11–17. 10.1016/j.jtbi.2013.07.009 23871866

[B41] UniProt Consortium T. (2018). UniProt: the universal protein knowledgebase. Nucleic Acids Res. 46 (5), 2699. 10.1093/nar/gky092 29425356 PMC5861450

[B42] Vacic V. Iakoucheva L. M. Radivojac P. (2006). Two Sample Logo: a graphical representation of the differences between two sets of sequence alignments. Bioinformatics 22 (12), 1536–1537. 10.1093/bioinformatics/btl151 16632492

[B43] Wang X. B. Wu L. Y. Wang Y. C. Deng N. Y. (2009). Prediction of palmitoylation sites using the composition of k-spaced amino acid pairs. Protein Eng. Des. Sel. 22 (11), 707–712. 10.1093/protein/gzp055 19783671

[B44] Wang L. N. Shi S. P. Xu H. D. Wen P. P. Qiu J. D. (2017). Computational prediction of species-specific malonylation sites via enhanced characteristic strategy. Bioinformatics 33 (10), 1457–1463. 10.1093/bioinformatics/btw755 28025199

[B45] Wei L. Xing P. Shi G. Ji Z. L. Zou Q. (2018). Fast prediction of protein methylation sites using a sequence-based feature selection technique. IEEE/ACM Trans. Comput. Biol. Bioinf. PP (99), 1–1. 10.1109/TCBB.2017.2670558 28222000

[B46] Xu J. He Y. Qiang B. Yuan J. Peng X. Pan X. M. (2008). A novel method for high accuracy sumoylation site prediction from protein sequences. BMC Bioinf. 9, 8. 10.1186/1471-2105-9-8 PMC2245905 18179724

[B47] Xu Y. Wang X.-B. Ding J. Wu L.-Y. Deng N.-Y. (2010). Lysine acetylation sites prediction using an ensemble of support vector machine classifiers. J. Theor. Biol. 264 (1), 130–135. 10.1016/j.jtbi.2010.01.013 20085770

[B48] Xu Y. Ding J. Huang Q. Deng N.-Y. (2013). Prediction of protein methylation sites using conditional random field. Protein Pept. Lett. 20 (1), 71–77. 10.2174/092986613804096865 22789108

[B49] Xu G. Wang J. Wu Z. Qian L. Dai L. Wan X. (2014). SAHA regulates histone acetylation, Butyrylation, and protein expression in neuroblastoma. J. Proteome Res. 13 (10), 4211–4219. 10.1021/pr500497e 25160476

[B50] Xu H. D. Shi S. P. Wen P. P. Qiu J. D. (2015a). SuccFind: a novel succinylation sites online prediction tool *via* enhanced characteristic strategy. Bioinformatics 31 (23), 3748–3750. 10.1093/bioinformatics/btv439 26261224

[B51] Xu Y. Ding Y. X. Ding J. Lei Y. H. Wu L. Y. Deng N. Y. (2015b). iSuc-PseAAC: predicting lysine succinylation in proteins by incorporating peptide position-specific propensity. Sci. Rep. 5, 10184. 10.1038/srep10184 26084794 PMC4471726

[B52] Xu Y. Ding J. Wu L. Y. (2016). iSulf-Cys: prediction of S-sulfenylation sites in proteins with physicochemical properties of amino acids. PloS One 11 (4), e0154237. 10.1371/journal.pone.0154237 27104833 PMC4841585

[B53] Xu H. Zhou J. Lin S. Deng W. Zhang Y. Xue Y. (2017). PLMD: an updated data resource of protein lysine modifications. J. Genet. Genomics 44 (5), 243–250. 10.1016/j.jgg.2017.03.007 28529077

[B54] Xu J. Y. Xu Z. Liu X. Tan M. Ye B. C. (2018). Protein acetylation and butyrylation regulate the phenotype and metabolic shifts of the endospore-forming *Clostridium acetobutylicum* . Mol. Cell Proteomics 17 (6), 1156–1169. 10.1074/mcp.RA117.000372 29523768 PMC5986239

[B55] Xue Y. Chen H. Jin C. Sun Z. Yao X. (2006). NBA-Palm: prediction of palmitoylation site implemented in naive Bayes algorithm. BMC Bioinf. 7, 458. 10.1186/1471-2105-7-458 PMC1624852 17044919

[B56] Zhang K. Chen Y. Zhang Z. Zhao Y. (2008). Identiﬁcation and veriﬁcation of lysine propionylation and butyrylation in yeast core histones using PTMap Software. J. Proteome Res. 8 (2), 900–906. 10.1021/pr8005155 PMC2921183 19113941

[B57] Zhang N. Li B.-Q. Gao S. Ruan J.-S. Cai Y.-D. (2012). Computational prediction and analysis of protein γ-carboxylation sites based on a random forest method. Mol. Biosyst. 8 (11), 2946–2955. 10.1039/c2mb25185j 22918520

[B58] Zhang W. Xu X. Yin M. Luo N. Zhang J. Wang J. (2013). Prediction of methylation sites using the composition of k-spaced amino acid pairs. Protein Pept. Lett. 20 (8), 911–917. 10.2174/0929866511320080008 23276225

[B59] Zhang Y. Tang L. Zou H. Yang Q. Yu X. Jiang J. (2015). Identifying protein arginine methylation sites using global features of protein sequence coupled with support vector machine optimized by particle swarm optimization algorithm. Chemom. Intell. Lab. Syst. 146, 102–107. 10.1016/j.chemolab.2015.05.011

[B60] Zhao X. Zhang W. Xu X. Ma Z. Yin M. (2012). Prediction of protein phosphorylation sites by using the composition of k-spaced amino acid pairs. PloS One 7 (10), e46302. 10.1371/journal.pone.0046302 23110047 PMC3478286

[B61] Zhao X. Dai J. Ning Q. Ma Z. Yin M. Sun P. (2013). Position-specific analysis and prediction of protein pupylation sites based on multiple features. BioMed. Res. Int. 2013, 109549. 10.1155/2013/109549 24066285 PMC3770009

[B62] Zhou F. F. Xue Y. Chen G. L. Yao X. (2004). GPS: a novel group-based phosphorylation predicting and scoring method. Biochem. Biophys. Res. Commun. 325 (4), 1443–1448. 10.1016/j.bbrc.2004.11.001 15555589

[B63] Zhou F. Xue Y. Yao X. Xu Y. (2006). CSS-Palm: palmitoylation site prediction with a clustering and scoring strategy (CSS). Bioinformatics 22 (7), 894–896. 10.1093/bioinformatics/btl013 16434441

[B64] Zhou Y. Huang T. Huang G. Zhang N. Kong X. Cai Y.-D. (2016). Prediction of protein N-formylation and comparison with N-acetylation based on a feature selection method. Neurocomputing 217, 53–62. 10.1016/j.neucom.2015.10.148

